# Self-assembled ferritin nanoparticles displaying PcrV and OprI as an adjuvant-free *Pseudomonas aeruginosa* vaccine

**DOI:** 10.3389/fimmu.2023.1184863

**Published:** 2023-06-21

**Authors:** Yuhang Li, Ruixue Pu, Yi Zhang, Yiwen Zhang, Yujie Wei, Sheng Zeng, Chen Gao, Ying Wang, Daijiajia Yin, Yueyue Zhang, Jiqing Wan, Quanming Zou, Jiang Gu

**Affiliations:** ^1^ College of Pharmacy, Dali University, Dali, China; ^2^ National Engineering Research Center of Immunological Products, Department of Microbiology and Biochemical Pharmacy, College of Pharmacy, Army Medical University, Chongqing, China; ^3^ The Third Outpatient Department, The General Hospital of Western Theater Command, Chengdu, China; ^4^ 953th Hospital, Xinqiao Hospital, Army Medical University, Shigatse, China; ^5^ Health Management Center, PLA Hangzhou Sanatorium, Hangzhou, China

**Keywords:** ferritin nanoparticles, adjuvant-free, *Pseudomonas aeruginosa*, PcrV, OprI, vaccine

## Abstract

**Introduction:**

Serious infections of *Pseudomonas aeruginosa* (PA) in hospitals and the emergence and increase of multidrug resistance have raised an urgent need for effective vaccines. However, no vaccine has been approved to date. One possible reason for this is the limited immune response due to the lack of an efficient delivery system. Self-assembled ferritin nanoparticles are good carriers of heterogeneous antigens, which enhance the activation of immunological responses.

**Methods:**

In this study, two well-studied antigen candidates, PcrV and OprI, were selected and connected to the ferritin nanoparticle by the Spytag/SpyCatcher system to generate the nanovaccine rePO-FN.

**Results:**

Compared to recombinant PcrV-OprI formulated with aluminum adjuvants, intramuscular immunization with adjuvant-free rePO-FN induced quick and efficient immunity and conferred protection against PA pneumonia in mice. In addition, intranasal immunization with adjuvant-free rePO-FN enhanced protective mucosal immunity. Moreover, rePO-FN exhibited good biocompatibility and safety.

**Discussion:**

Our results suggest that rePO-FN is a promising vaccine candidate, as well as, provide additional evidence for the success of ferritin-based nanovaccines.

## 1 Introduction

*Pseudomonas aeruginosa* (PA) is a gram-negative opportunistic pathogen that causes pneumonia in patients on ventilators and patients with chronic obstructive pulmonary disease (COPD), cystic fibrosis, and other impaired respiratory function conditions (1). PA infections are also commonly found in hospitalized patients in intensive care and burn units (2). Currently, the application of antibiotics, such as aminoglycosides, quinolones, and beta-lactam antibiotics, is still of limited significance for reducing morbidity and mortality. Moreover, the emergence of multidrug-resistant strains of PA is increasing, making this problem more serious (3). Therefore, in 2019, PA was designated a critical priority for the development of new therapeutics by the World Health Organization. Vaccines can prevent infections and subsequently reduce the amount and frequency of antibiotics, resulting in the reduction of antibiotic resistance. Nevertheless, no licensed PA vaccine is available to date (4).

PA has a needle-like type III secretion system (T3SS) (5). PcrV is located at the needle end of T3SS and is involved in the translocation of toxins into host cells. PcrV is a well-conserved protein, with 98% homology among different isolates (6). Because of its critical contribution to pathogenesis and location on the cell membrane, PcrV is a promising candidate target for vaccine development. Several studies have shown that vaccines containing PcrV or its derivatives elicit protection in mice (7, 8). In addition, the anti-PcrV monoclonal antibody KB001-A has been tested in a phase 2 clinical trial (9). OprI of PA is a membrane lipoprotein that plays an important role its membrane integrity (10). Immunization with recombinant OprI conferred protection in mice. To date, the recombinant PA vaccine IC43, which has undergone a complete phase 2/3 clinical trial, consists of OprI and the outer membrane protein OprF (11). Moreover, OprI acts as an adjuvant when fused with other heterologous proteins (12).

Compared to monomeric soluble antigens, ordered antigens displayed on nanoparticles are favorable for the uptake of dendritic cells (DCs) and transport to lymph nodes, and subsequently enhance the activation of immunological responses (13). Ferritin is a biocompatible, biodegradable, and thermally stable nanoparticle that consists of 24 self-assembled subunits. It is found in almost all organisms. With multiple heterologous antigen insertion regions, ferritin nanoparticles also offer many modification selections, making them a promising platform for antigen presentation (14). For example, the SARS-Cov-2 RBD induces a persistent antibody response and long-term memory when delivered by ferritin nanoparticles (15).

**Figure 1:**
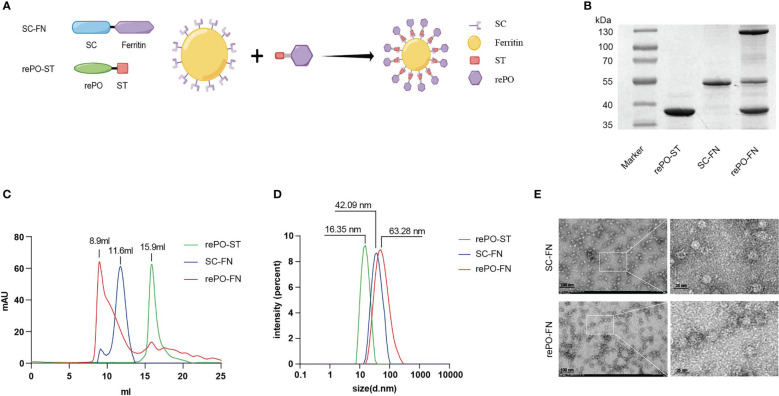
Production and characterization of rePO-Ferritin. **(A)** Schematic of components of rePO-Ferritin. SC, SpyCatcher; ST, SpyTag; rePO, PcrV-OprI; FN, Ferritin; Generated using Figdraw. **(B)** SDS-PAGE analysis of purified rePO-ST, SC-FN, and rePO-FN. **(C)** Characterization of rePO-ST, SC-FN, and rePO-FN by Superose 6 size-exclusion chromatography. **(D)** Characterization of rePO-ST, SC-FN, and rePO-FN by dynamic light scattering. **(E)** Images of SC-FN and rePO-FN nanoparticles by transmission electron microscopy (TEM). The scale bar represents 100 nm.

To develop an effective vaccine, we speculated that delivering PcrV/OprI using ferritin nanoparticles would improve its immunogenicity and protective effects. Therefore, in this study, we fused SpyCatcher to the N-terminus of ferritin to generate a vector containing candidate antigens (16). SpyTag was added to the C-terminus of the recombinant Pcrv-OprI (rePO). The SpyTag-tagged rePO was covalently connected to the surface of ferritin through the formation of an isopeptide bond between SpyTag and SpyCatcher (16), which generated the nanoparticle vaccine rePO-ferritin (rePO-FN). The immunogenicity and immunoprotective effects were evaluated in a mouse model (**Figure 1A**).

## 2 Materials and methods

### 2.1 Ethics statement

All the experiments were performed in compliance with the Laboratory Animal Management Rules of the People’s Republic of China. All animal care and experiments were approved by the Animal Ethics and Experimental Committee of the Army Military Medical University, China (No. AMUWEC20223337).

### 2.2 Bacterial strains and animals

PA XN-1 (CCTCC M2015730) (8) was isolated from a patient with severe pneumonia from Southwest Hospital in China. It was deposited at the China Center for Type Culture Collection (CCTCC). Female 6–8-week-old BALB/C mice were purchased from Beijing Vital River Laboratory Animal Technology Co., Ltd. and kept in a Specific-Pathogen-Free Animal Facility.

### 2.3 Cloning, expression, and purification of recombinant proteins

The sequences encoding PcrV (28–294) and OprI (25–83) were obtained from the *Pseudomonas aeruginosa* PAO1 genome. The SpyCatcher-tagged ferritin (SC-FN) and SpyTag-tagged rePO (rePO-ST) were cloned into the bacterial expression vectors pET28a and pET32a. Recombinant gene sequences were synthesized and provided by Genescript Biotechnology Ltd. Recombinant protein expression and purification of rePO-ST and SC-FN.DNA sequencing verified the construct, and the plasmid was transformed into *E. coli* BL21 competent cells. Single colonies were picked and inoculated into 10 mL Luria-Bertani (LB) broth with kanamycin (25 μg/ml) or ampicillin (50 μg/ml) and incubated overnight at 37°C at 180 rpm. The culture was then expanded to 2 L and induced with 0.2 mM of Isopropyl ß-D-1-thiogalactopyranoside (IPTG) and then incubated overnight at 37°C at 180 rpm. Cells were harvested by centrifugation, washed several times, and resuspended in sterile PBS (50 mM Tris-HCl, pH 8.5, 150 mM NaCl). The homogenate was lysed by ultrasonication on ice, followed by centrifugation to remove insoluble material. After centrifugation, the supernatant was purified using a nickel column. For the production of rePO-FN, SC-FN, and rePO-ST were mixed overnight at 4°C in a series of molar ratios (1:8, 1:12, 1:16, 1:20, 1:24 and 1:28, respectively). The products were then analyzed by SDS-PAGE to determine the optimal reaction ratio. Finally, protein samples were stored at -80°C.

### 2.4 Gel-filtration and dynamic light scattering assay

The molecular weight sizes of the fusion protein rePO-ST, SC-FN, and rePO-FN were determined separately using superose6 (Increase 10/300 GL) gel filtration chromatography. 500 µg of purified recombinant proteins (500 µL) were separately loaded onto the column, and the relative molecular masses were calculated using the corresponding elution volumes. Samples were diluted to 0.5 mg/mL, loaded onto a dynamic light scattering instrument, and analyzed on three separate occasions at 25 °C.

### 2.5 Electron microscopy

Electron microscopy analysis of the nanoparticles presented by SC-FN and rePO-FN was performed. Both sample concentrations were diluted to 0.01 to 0.05 mg/mL using a buffer (50 mM Tris-HCl, pH 8.5,150 mM NaCl). Carbon-coated copper grids (400 mesh) were glow-discharged, and 8 μL of each sample was adsorbed for 2 min. Excess samples were wicked away and grids were negatively stained with 2% uranyl formate for 2 min. When the grids were dry, the samples were analyzed using a transmission microscope at 80 kV and images were recorded.

### 2.6 Immunization of mice

To assess the effect of the intramuscular immune response, BALB/c mice were divided into four groups (PBS, rePO, rePO+Al(OH)_3_, and rePO-FN). A volume of 0.2 mL of each preparation was intramuscularly injected into the four groups of mice on days 0, 7, and 14. For molar equivalent doses, each injection contains 10 µg of rePO or 29.2 µg of rePO-FN, which contains 10 µg of rePO and 19.2 μg of ferritin.To examine the immune response, BALB/c mice were divided into three groups (PBS, rePO, and rePO-FN) for intranasal administration. The mice were anesthetized using 0.14 mL (10 mg/mL) of sodium pentobarbital intraperitoneally and were immunized by placing 12.5 μL of the vaccine inocula into each nasal cavity. Same amount of antigen was used as that for intramuscular immunization. Mice were immunized on days 0, 14, and 21. Meanwhile, Mice immunized with SC-FN alone in the same way were also used as controls.

### 2.7 Murine pneumonia model

For the acute pneumonia model, mice in each group were anesthetized with pentobarbital sodium, followed by injection of a lethal dose (1.17×10^7^ CFU per mouse) of PA XN-1. Mice were observed for body weight, signs of infection, and death every 12h after the challenge for 7 days. Other mice were injected with a sublethal dose (2.94×10^6^ CFU per mouse) of PA XN-1 to evaluate other indicators. The global disease score of each mouse was defined as we used before with slight modification (17). In brief, after challenge with sublethal dose five mice in each group were randomly selected for observation, and the mice were observed for physical signs of infection every 12 h. The observation indexes and scoring criteria were as follows: breathing, piloerection, movement, nasal secretion and posture. Then, the global score was recorded as unaffected (0–1), slightly affected (2–4), moderately affected (5–7), or severely affected (8–10). To minimize bias, all mice were scored by the same two researchers in a blinded manner.

### 2.8 Enzyme-linked immunosorbent assay

Sera were collected from immunized mice one week after each immunization. Seven days after the last immunization, the nasal lavage fluid (NALF) and bronchoalveolar lavage fluid (BALF) were collected. 96-well ELISA plates were coated with 6 μg/mL rePO protein in carbonate buffer (100 μL/well) overnight at 4°C. After blocking with 1% of bovine serum albumin, diluted samples were incubated for 1 h at 37°C. Goat anti-mouse IgA-HRP (1:7500) or goat anti-mouse IgG-HRP (1:7500) were used as the detection antibodies. Absorbance was read at 450 nm using a microplate reader.

### 2.9 Enzyme-linked immunospot assay

The Mouse IL-17A (1) ELISpot Kit (MABTECH) was used to detect peptide-specific IL-17-producing cells and the IFN-γ ELISpot Kit (MABTECH) was used to detect IFN-γ secretion. To obtain splenic single-cell suspension, the mouse spleen was ground and filtered. Then cells were plated at a cell concentration of 5×10^5^ cells/well. The experimental group was stimulated with rePO peptide pools and the control group was stimulated with DMSO. After 48 h of incubation, the ELISPOT assay was performed according to the manufacturer’s instructions.

### 2.10 Bacterial burden

Lung tissue was collected 18 h after the challenge. The tissues were homogenized in 1 mL of sterile PBS and serially diluted. Homogenates at a 10-fold dilution gradient were dropped onto a solid LB plate. After incubation at 37°C for 20 h, the total number of bacteria in the lungs was determined.

### 2.11 Histological analysis

Lung tissue was collected from mice 18 h after infection, fixed in 4% paraformaldehyde, paraffin-embedded, sectioned, and stained with hematoxylin-eosin (HE). The sections were viewed at 100 × and 400 × magnification. Each lung section was assigned a score of 0-8 (no abnormality to most severe) according to the established criteria based on congestion, edema, hemorrhage, and neutrophil infiltration.

### 2.12 Evaluation of inflammation

The lungs of the mice were collected 18 h after challenge with a sub-lethal dose of PA XN-1. The lung tissue was sonicated, and the supernatant was centrifuged and diluted to a suitable multiple in sterile PBS. TNF-α, IL-1β, IL-6, and IL-12 levels were measured separately using kits (DAKEWE) according to the manufacturer’s instructions to determine the concentration of pro-inflammatory cytokines.

### 2.13 Cytolytic assay

Fresh blood was collected from mice to obtain red blood cells, which were diluted to a concentration of 2% (v/v) in sterile saline. In each tube, 100 μL of 2% erythrocyte suspension was added, followed by 900 μL of rePO-FN protein at 25, 50, 75, and 100 μg/mL. Saline and distilled water were used as the negative and positive controls, respectively. After incubation for 2 h at 37°C, samples were centrifuged, and the absorbance of the supernatant was measured at 545 nm.

### 2.14 CCK8 assay

The CCK8 assay was conducted using the CCK8 kit (DAKEWEI) as described previously (18). In brief, 100 μL of DC2.4 cell suspension (1×10^6^/mL) was prepared in a 96-well plate. The test sample was added and the plates were incubated for 24 h. Finally, 10 μL of the CCK8 solution was added to each well. The plates were incubated for 3 h and the absorbance at 450 nm was measured using an enzyme marker.

### 2.15 *In vivo* safety evaluation

Mice were immunized with rePO-FN thrice, as described in the previous section. One week after the last immunization. The heart, liver, spleen, lungs, and kidneys were collected, fixed in 4% paraformaldehyde, embedded in paraffin, sectioned, and stained with HE. Sections were observed at 100 × magnification.

### 2.16 Statistical analysis

Data are presented as the means ± Standard Deviation (SD). The scoring experiments were performed in a blinded manner. The log-rank test, Student’s t-test, and one-way ANOVA with Bonferroni correction were used, depending on the sample distribution and variation. GraphPad Prism 6.0 (GraphPad Software, Inc., USA) was used to perform statistical analyses. Statistical significance was set at *P* < 0.05.

## 3 Results

### 3.1 Production and characterization of rePO-Ferritin

As shown in **Figure 1**, both SpyTag-tagged rePO (rePO-ST) and nanocarrier SpyCatcher-tagged ferritin (SC-FN) were successfully produced in soluble form in *E. coli* and purified by Ni-NTA affinity chromatography. The purities of rePO-ST and SC-FN were approximately 93.2% and 91.2%, respectively, when analyzed by SDS-PAGE. Then the SC-FN and rePO-ST were co-incubated *in vitro* at 4°C for 12 hours. As expected, rePO-ST efficiently conjugated to the carrier SC-FN, generating the nanovaccine rePO-FN represented as a new band on SDS-PAGE (**Figure 1B**). To optimize the reaction, a molar gradient ratio of rePO-ST and SC-FN, namely 1:8, 1:12, 1:16, 1:20, 1:24 and 1:28, was tested. The proportion of unconjugated ferritin decreased when the ratio increase. However, no apparent change of unconjugated ferritin was noted when the molar ratio was over 1:20 (**Figure S1**). As a result, the ratio of 1:20 was applied for the following rePO-ST and SC-FN conjugation experiments. In addition, the result from size exclusion chromatography (SEC) showed that the rePO-FN forms a major peak at 8.9 mL, while the elution volume of SC-FN and rePO-ST was 11.6 and 15.9 mL, respectively (**Figure 1C**). The dynamic light scattering assay showed that the SC-FN forms a uniform peak at a diameter of approximately 42.09 nm, while rePO-FN forms the major peak at 63.28 nm (**Figure 1D**). Moreover, both rePO-FN and SC-FN formed nanoparticles with a diameter of 17.74 nm and 30.39 nm when observed using electron microscopy, respectively (**Figure 1E**).

### 3.2 Intramuscular immunization of adjuvant-free rePO-FN induced quick and efficient immunity

**Figure 2:**
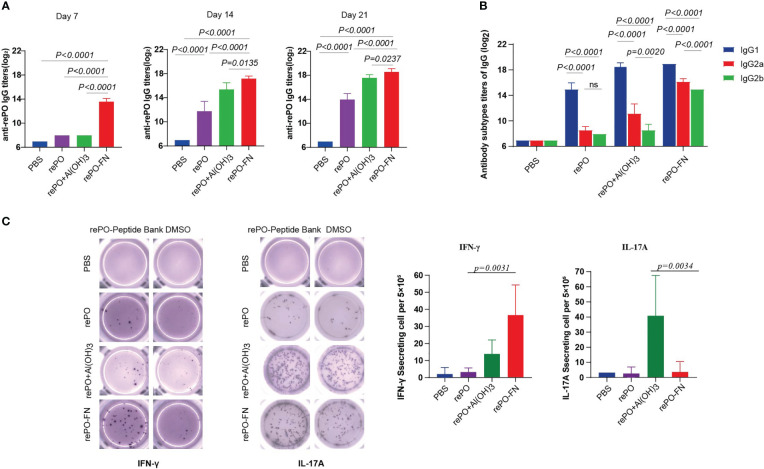
Intramuscular immunization of adjuvant-free rePO-FN induced quick and efficient immunity. **(A)** The bar represents the titer of anti-rePO IgGs in the sera of immunized mice (n = 5). Sera were collected on day7, 14, and 21. **(B)** The subtypes of anti-rePO IgGs in the sera of immunized mice. The bar represents the titer of anti-rePO IgG1, IgG2a, and IgG2b (n = 5). **(C)** Cellular immune responses in spleen cells. The numbers of IFN-γ and IL-17A-secreting cells from the spleen were determined by ELISPOT (n = 5). Statistical significance was set at *P* < 0.05.

First, the levels of anti-rePO IgG in mice immunized with adjuvant-free rePO-FN and rePO were determined. The rePO formulated with Al(OH)_3_ was used as a control. The titer of only rePO-FN-immunized mice significantly increased as early as 7 days after the first immunization (**Figure 2A**). In addition, the rePO-FN-immunized group had higher anti-rePO IgG titers than that of the free rePO or that formulated with rePO formulated with Al(OH)_3_. Consistent with our previous study, the application of Al(OH)_3_ enhanced the immunogenicity of rePO (**Figure 2A**). The predominant subtypes of IgGs in the three immunization groups were all IgG1, suggesting a Th2-predominant immune response (**Figure 2B**). Meanwhile, an evident increase of anti-ferritin antibodies was observed in mice immunized with SC-FN and rePO-FN (**Figure S2A**). In addition, results from the enzyme-linked immunospot (ELISPOT) assay showed that the number of IFN-γ-secreting lymphocytes drastically increased only in the spleen of rePO-FN-immunized mice (**Figure 2C**), suggesting a Th1 response. Interestingly, a significant increase in IL-17A-secreting lymphocytes was observed in the mice immunized with the rePO formulated with Al(OH)_3_ (**Figure 2C**). Collectively, these data indicated that adjuvant-free rePO-FN induced quick and efficient Th1 and Th2 responses.

### 3.3 Intramuscular immunization of adjuvant-free rePO-FN conferred protection against pneumonia in mice

**Figure 3:**
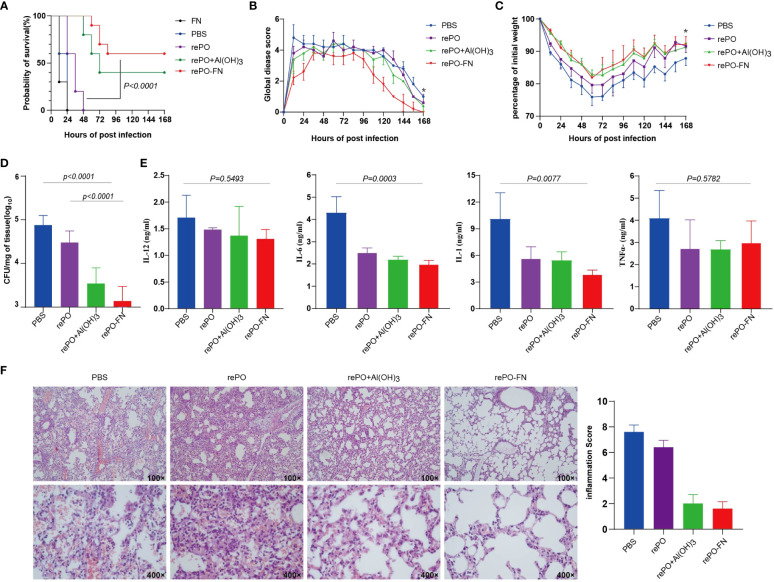
Intramuscular immunization of adjuvant-free rePO-FN induced quick and efficient immunity. **(A)** The intramuscularly immunized mice were challenged by an intratracheal injection of PA XN-1. The survival rates of the mice immunized with rePO-FN, adjuvant-free rePO and FN, Al(OH)_3_ formulated rePO (n = 10). **(B)** The global disease score of immunized mice after challenge with a sublethal dose of PA XN-1 (n = 5). The score was recorded every 12 h for 7 days. **(C)** Weight loss in immunized mice after challenge with a sublethal dose of PA XN-1 (n = 5); the weight was recorded every 12 h for 7 days. **(D)** Assessment of the bacterial load in the spleen of immunized mice 18 h after the challenge with a sublethal dose of PA XN-1. The bar represents the log CFU per mg of the spleen (n = 5). **(E)** Semi-quantitative measurement of the pro-inflammatory cytokines TNF-α, IL-6, IL-12p, and IL-1β in mice lungs (n = 3). **(F)** HE staining of lungs. The immunized mice were challenged with a sublethal dose of PA XN-1. 18 hours later, the lung was collected and stained with hematoxylin and eosin. Images were captured at 100× magnification and 400× magnification. Statistical significance was set at *P* < 0.05.

To evaluate the protection of rePO-FN, intramuscularly immunized mice were challenged with an intratracheal injection of a lethal dose of PA XN-1 (1.17× 10^7^ CFU/mL per mouse). As shown in **Figure 3A**, the survival rate of the free rePO-FN-immunized group was 60%, which was significantly higher than that in the other four groups. Clearly, no protection was observed in FN immunized group. To further investigate the mechanism of rePO-FN-induced protection, the immunized mice were challenged with a sublethal dose of PA XN-1 (2.94 × 10^6^ CFU/mL per mouse). The global disease score of mice in the rePO-FN-immunized group peaked at 72 h after infection and decreased immediately. The area under the curve (AUC) of the rePO-FN group was significantly smaller than that of the other three groups, indicating moderate infection in mice (**Figure 3B**). In addition, rePO-FN and Al(OH)_3_-formulated rePO immunized mice showed a slower weight loss and a faster recovery (**Figure 3C**). Moreover, the amount of bacterial colonization in the lungs and spleens of the rePO-FN-immunized mice was significantly lower than that in those immunized with free rePO and Al(OH)_3_-formulated rePO (**Figure 3D**). A similar trend in the concentration of the pro-inflammatory cytokines TNF-α, IL-6, IL-12p, and IL-1β in the lung was also observed (**Figure 3E**). Histological changes in the lungs, such as congestion, edema, inflammatory cell infiltration, and alveolar cell rupture, were observed in the PBS control group, indicating severe pneumonia. However, these changes were significantly weaker in rePO-FN-immunized mice (**Figure 3F**). Taken together, immunization with adjuvant-free rePO-FN reduced bacterial colonization, inflammation, and damage in the lungs and subsequently protected mice against PA challenge.

### 3.4 Intranasal immunization of rePO-FN enhances mucosal immunity

**Figure 4:**
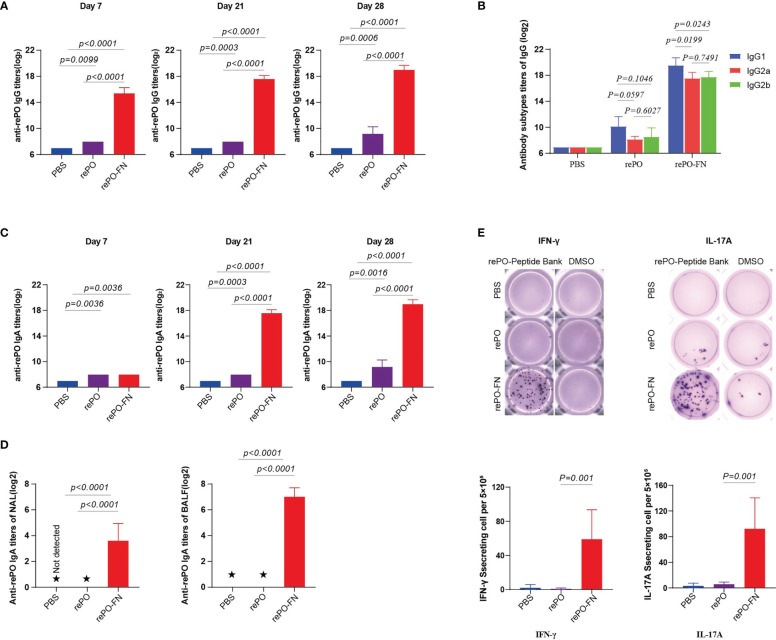
Intranasal immunization of rePO-FN enhances mucosal immunity. **(A)** The bar represents the titer of anti-rePO IgGs in the sera of immunized mice (n = 5). Sera were collected on day7, 21, and 28. **(B)** Subtypes of anti-rePO IgGs in the sera of immunized mice. The bar represents titers of anti-rePO IgG1, IgG2a, and IgG2b (n = 5). **(C)** The bar represents the titer of anti-rePO IgAs in the sera of immunized mice (n = 5). Sera were collected on day7, 21, and 28. **(D)** The bar represents the titer of anti-rePO IgAs in the bronchoalveolar lavage fluid (BALF) and nasal lavage fluid (NLF) of immunized mice (n = 5). **(E)** Cellular immune response in spleen cells. The number of IFN-γ- and IL-17A-secreting cells in the spleen was determined by ELISPOT (n = 5). Statistical significance was set at *P* < 0.05. ★, the potency of the antibody in the lavage solution was too low to detect a valid value.

These findings led us to test whether intranasal immunization with rePO-FN induced mucosal immunity in the lungs where PA was colonized. First, we tested anti-rePO IgGs in the sera of mice after intranasal immunization. Surprisingly, the titer of anti-rePO IgGs of rePO-FN was the highest among the three groups on days 7, 21, and 28 (**Figure 4A**). Similar to intramuscular immunization, IgG1 accounted for the main subtypes of IgGs after intranasal immunization with both rePO and rePO-FN (**Figure 4B**). More importantly, the titer of serum IgAs in the rePO and rePO-FN groups increased as early as 7 days after the first immunization. Compared to the rePO group, a dramatic increase in anti-rePO serum IgA levels was observed in the rePO-FN group on days 21 and 28 (**Figure 4C**). Likewise, anti-rePO IgAs in the NAL and BALF were found only in the rePO-FN group (**Figure 4D**). Meanwhile, the ELISPOT assay showed that the number of IFN-γ-secreting lymphocytes in the spleen dramatically increased only in the rePO-FN- group. Surprisingly, the number of IL-17A-secreting lymphocytes also significantly increased after intranasal immunization, which was different from that seen after intramuscular immunization (**Figure 4E**). In addition, the titers of anti-ferritin antibodies in SC-FN and rePO-FN immunized mice increased significantly on day 7, day 14 and day 28 (**Figure S2B**).

### 3.5 Intranasal immunization of adjuvant-free rePO-FN conferred protection against pneumonia in mice

**Figure 5:**
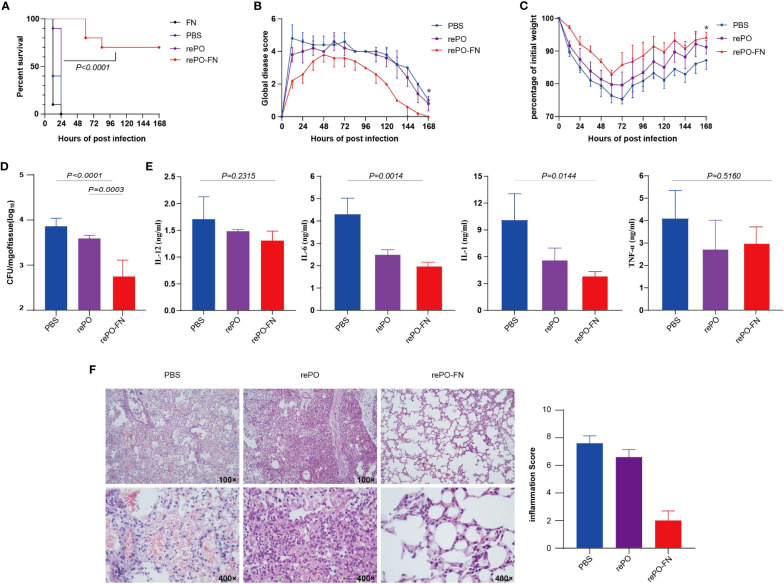
Intranasal immunization of adjuvant-free rePO-FN conferred protection against pneumonia in mice. **(A)** The intranasally immunized mice were challenged by an intratracheal injection of PA XN-1. The survival rates of the mice immunized with rePO-FN and adjuvant-free rePO and FN (n = 10). **(B)** The global disease score of immunized mice after challenge with a sublethal dose of PA XN-1 (n = 5). The score was recorded every 12 h for 7 days. **(C)** Weight loss in immunized mice after challenge with a sublethal dose of PA XN-1 (n = 5); the weight was recorded every 12 h for 7 days. **(D)** Assessment of the bacterial load in the spleen of immunized mice 18 h after challenge with a sublethal dose of PA XN-1. The bar represents the log number of CFU per mg of the spleen (n = 5). **(E)** Semi-quantitative measurement of the pro-inflammatory cytokines TNF-α, IL-6, IL-12p, and IL-1β in mice lungs (n = 3). **(F)** Hematoxylin and eosin staining of lungs. The immunized mice were challenged with a sublethal dose of PA XN-1. 18 h later, the lungs were collected and stained with hematoxylin and eosin. Images were captured at 100× and 400× magnification. Statistical significance was set at *P* < 0.05.

These findings led us to evaluate the protective effects of rePO-FN intranasal immunization. As expected, the survival rate of the rePO-FN group was 100%, which was significantly higher than that of the rePO group. However, no survival was observed 24 hours post the challenge in SC-FN immunized mice (**Figure 5A**). When the immunized mice were challenged with a sub-lethal dose of PA XN-1, the global disease score and body weight suggested that intranasal immunization with rePO-FN significantly reduced PA infection (**Figure 5B, C**). In addition, the number of colonized bacteria in the lungs decreased significantly in the rePO-FN-immunized mice (**Figure 5D**). Consistently, the concentrations of the pro-inflammatory cytokine TNF-α, IL-6, IL-12p, and IL-1β in the lungs of rePO-FN-immunized mice were significantly lower than those of the other two groups (**Figure 5E**). Observations of histological changes confirmed this trend (**Figure 5F**). Taken together, these data demonstrated that intranasal immunization with adjuvant-free rePO-FN conferred protection against pneumonia in mice.

### 3.6 rePO-FN is of good biocompatibility and safety

**Figure 6:**
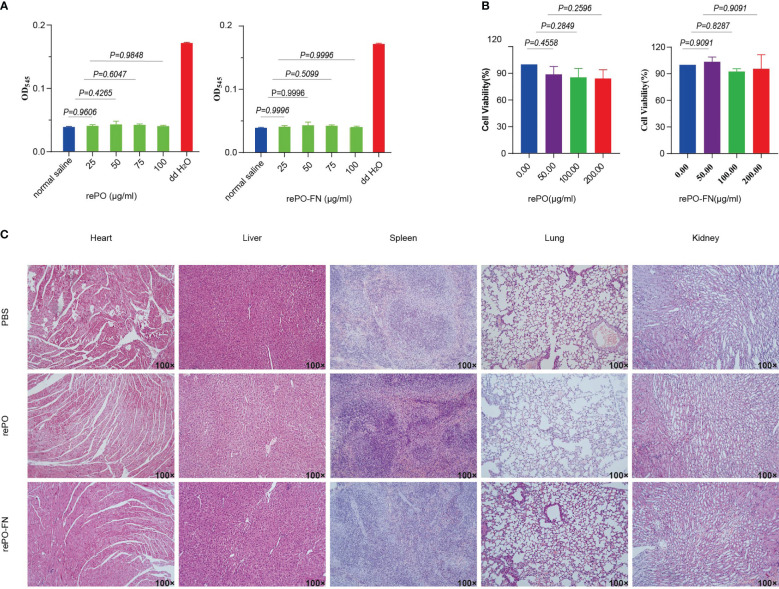
The rePO-FN is of good biocompatibility and safety. **(A)** Hemolytic activity of rePO-FN protein. **(B)** The bar represents the cell viability as determined by the CCK-8 assay. **(C)** Representative images of sections of the heart, liver, spleen, lung, and kidney after HE staining.

First, we performed an *in vitro* cytolytic assay to evaluate the biocompatibility of rePO-FN. As shown in **Figure 6A**, the hemolysis rate did not increase significantly compared to the PBS control, even at concentrations of rePO-FN and rePO as high as 100 μg/mL. The results from the CCK8 assay showed that rePO-FN and rePO did not significantly affect cell viability (**Figure 6B**). In addition, rePO-FN immunization of mice did not cause any significant damage to the heart, liver, spleen, lung, and kidney organs, as detected by histological analysis (**Figure 6C**). Thus, rePO-FN exhibited good biocompatibility and safety.

## 4 Discussion

Several years have passed since PA was first identified as a causative pathogen. Many protective antigens, including PcrV, OprF, OprI, and FlgE have been tested in recent years (4). In particular, vaccines targeting lipopolysaccharide, flagellin, and the outer membrane protein OprF/I have been evaluated in clinical trials (11). However, no PA vaccine has been approved to date. One of the main reasons for this is the insufficient speed and efficacy of the immune response induced by the existing vaccines. In this study, we chose the key component proteins of the T3SS secretion system, PcrV, and the outer membrane protein, OprI, as antigens. To enhance immunogenicity, the two antigens were attached to the surface of the ferritin particles, generating the nanoparticle vaccine rePO-FN. Adjuvant-free rePO-FN significantly enhanced the speed and protective efficiency of the immune response when immunized *via* either the intramuscular or intranasal route. In addition, molecular epidemiological studies have revealed that PcrV and OprI are highly conserved in clinical PA strains. Collectively, these results suggest that rePO-FN is a promising vaccine candidate.

The selection of protective antigens is critical in the development of bacterial vaccines. PcrV is located at the end of the bacterial pinpoint type III secretion system and is responsible for the delivery of bacterial toxins to host cells (19). Previous studies have shown that anti-PcrV antibodies block the secretion of toxins (20). In addition, they enhance the phagocytic activity of macrophages and promote the internalization of PA, facilitating bacterial clearance (21). Notably, OprI is a conserved outer membrane protein. It enhances the immunogenicity of fused antigens by promoting the production of IL-4, IL-6, IL-10, IFN-γ, TNFα, and IgGs (8). Therefore, it is not surprising that rePO-FN was highly protective.

Currently, there are two major strategies for connecting antigens to nanoparticle vectors. One is direct fusion at the gene level and the expression of the recombinant nanoparticle vaccine (22). The fusion of antigens and vectors usually affects protein folding, which limits the production of recombinant vaccines. Another strategy is to produce the antigen and nano-vector separately and then covalently or non-covalently connect them. SpyTag/SpyCatcher is a popular covalent coupling method owing to its high efficiency (22). Using the SpyTag/SpyCatcher system, the RBD of SARS-CoV-2 was coupled to virus-like particles. This nanoparticle vaccine induced a strong neutralizing antibody response, which was superior to that of the recovered patient sera (15). In the present study, Spy-tagged rePO was also efficiently coupled to ferritin protein nanoparticles, providing additional evidence that SpyTag/SpyCatcher is an efficient antigen-conjugation system for nanovaccines.

The immunostimulatory effects of aluminum adjuvants are widely accepted; however, there is still a safety concern because aluminum adjuvants are associated with the activation of the NLRP3 inflammatory vesicle and Syk-PI3 kinase pathways (21, 22). One possible solution to improve the immunogenicity of protein antigens without aluminum adjuvants is the application of soluble control peptide (SCP) tags. The immunogenicity of SCP-tagged ED3 (dengue envelope protein structural domain 3) was significantly higher than that of the monomeric form (23). The immune response of adjuvant-free HSV-1 (24) (herpes simplex virus type 1) 1 and HPV16 (25) (human papillomavirus 16) was enhanced after fusion with T4 phage nanoparticles. 3MCD-ferritin is also a nanoparticle carrying M2e and CD helix. Subcutaneous immunization with adjuvant-free 3MCD-ferritin induced Th1 and Th2 immune responses and provided complete protection against the H3N2 virus (26). In our study, without any adjuvant, the level of immune response induced by rePO-FN was as high as that induced by rePO formulated with Al(OH)_3_. Taken together, the delivery of antigens using ferritin or other nanoparticles is one of the strategies to avoid the use of aluminum adjuvants to address its safety concerns.

Traditionally, aluminum adjuvants facilitate the induction of a Th2-based immune response (26, 27). However, recent findings indicate that it can also induce a Th17 response. For example, a recombinant yeast protein with aluminum hydroxide (AH) induced protection against *Staphylococcus aureus* and *Candida albicans*. This protection depends on IL-17A and the induction of Th17 cells in the draining lymph nodes (27). Our previous study also found that PcrV_NH_ and POH formulated with aluminum adjuvant-induced Th1, Th2, and Th17 responses (17). In the present study, we found that compared to adjuvant-free rePO, AH-adsorbed rePO significantly increased the number of IL- 17A-secreting cells in splenocytes. These findings reinforce that aluminum adjuvants can induce a Th17 response. A possible explanation for this is the presence of inflammasome-derived IL-1 and IL-18 in response to aluminum adjuvants (28). However, the detailed mechanism requires further investigation.

The local mucosal immune response is a key barrier against lung infection. Thus, vaccines that induce lung mucosal immunity usually achieve significantly enhanced protection. The approved influenza vaccine Flumist is a good example of the induction of mucosal immunity and has shown a rapid and broad protective capacity (29). In the case of PA vaccines, intranasal immunization with recombinant PopB-PcrH and OprL induced a strong pulmonary mucosal Th17 response and protected against several clinical isolates of PA strains (30, 31). In addition, PcrV and AmpC-based multi-epitope vaccine PVAC mixed with the mucosal adjuvant Curdlan significantly enhanced protection against pulmonary pathogens by inducing pulmonary tissue-resident memory T cells (7). This explains why the protection achieved by rePO-FN *via* the intranasal route was significantly greater than that obtained *via* the intramuscular pathway. Future studies will focus on the mechanism of rePO-FN-induced mucosal immunity and its contribution to vaccine-induced protection.

One limitation of our study is that the conjugation between rePO-ST and SC-FN is not entirely effective. The proportion of conjugated ferritin remain about 70% and unchanged even excessive rePO was introduced. The reason could be fusion of SpyTag to rePO affects its structure, and subsequently reduces the conjugating efficiency. One possible solution is to optimize the reaction to produced more conjugated products or to purify the products using size-exclusion or other chromatography methods.

In conclusion, we successfully constructed a promising nanoparticle vaccine, rePO-FN. Adjuvant-free rePO-FN significantly enhanced the speed and protective efficiency of the immune response when immunized by either the intramuscular or intranasal route.

## Data availability statement

The original contributions presented in the study are included in the article/**Supplementary Material**. Further inquiries can be directed to the corresponding authors.

## Ethics statement

All the experiments were performed in compliance with the Laboratory Animal Management Rules of the People’s Republic of China. All animal care and experiments were approved by the Animal Ethics and Experimental Committee of the Army Military Medical University, China (No. AMUWEC20223337).

## Author contributions

YL: Data curation, Writing - original draft. RP: Data curation. YZ: Data curation. YWZ: Data curation. YJW: Data curation. SZ: Data curation. CG: Methodology, Software. YW: Methodology, Software. YYZ: Methodology, Software. JW: Methodology, Software. QZ: analyzed the data and revised the manuscript. JG: designed the experiments and got the grants. All authors contributed to the article and approved the submitted version.
