# Supplementary material for: Self-assembled ferritin nanoparticles displaying PcrV and OprI as an adjuvant-free Pseudomonas aeruginosa vaccine
Source: Front Immunol. 2023 Jun 21;14:1184863. doi: 10.3389/fimmu.2023.1184863 (PMC10321299; doi:10.3389/fimmu.2023.1184863)
Supplement: Supplementary file 1 [file DataSheet_1.zip › Supplementary Figure 1.DOCX]

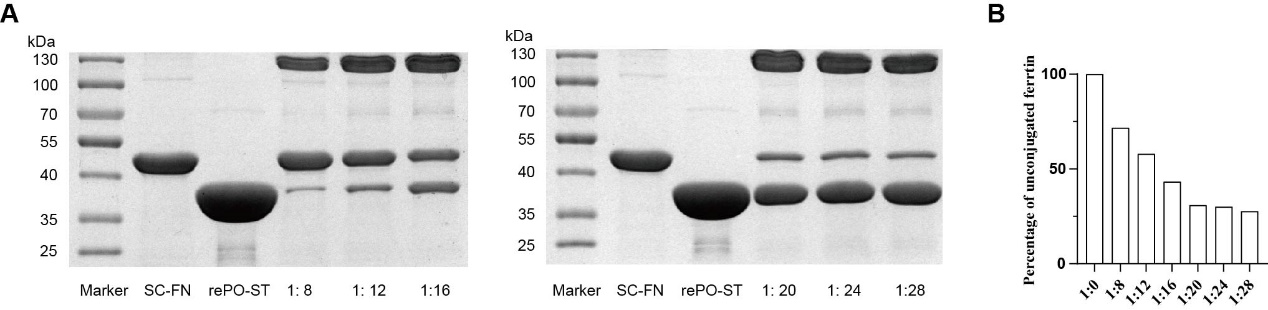


**Figure S1 Optimization of the conjugating of rePO-ST with SC-FN.** (A) SDS-PAGE analysis of products after incubation of SC-FN and rePO-ST at 4 °C for 12 hours at a molar ratio of 1:8, 1:12, 1:16, 1:20, 1:24, 1:28, respectively. (B) The percentage of unconjugated ferritin after the reaction. Data were from the SDS-PAGE gels and analyzed by ImageJ software.
