# Supplementary material for: Self-assembled ferritin nanoparticles displaying PcrV and OprI as an adjuvant-free Pseudomonas aeruginosa vaccine
Source: Front Immunol. 2023 Jun 21;14:1184863. doi: 10.3389/fimmu.2023.1184863 (PMC10321299; doi:10.3389/fimmu.2023.1184863)
Supplement: Supplementary file 1 [file DataSheet_1.zip › Supplementary Figure 2.DOCX]

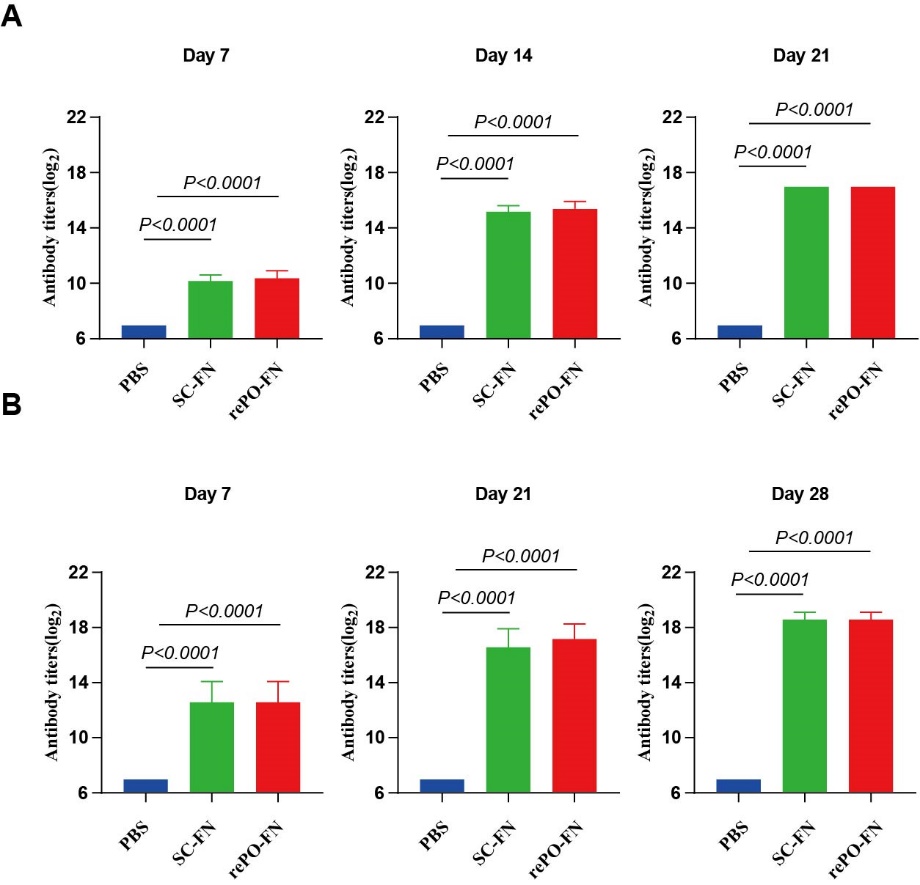


**Figure S2. The levels of anti-ferritin IgGs in mice immunized with rePO-FN and ferritin alone (SC-FN).** (A) Bar represents the titer of anti-ferritin total IgGs in the sera of intramuscularly immunized mice on day 7, day 14 and day 21. (B) Bar represents the titer of anti-ferritin total IgGs in the sera of Intranasally immunized mice on day 7, day 14 and day 28. Data are presented as the mean ± SE.
